# Safety in the transport of substances of human origin: a critical challenge for the quality of transplants

**DOI:** 10.1590/1516-3180.2026.1443.23022026

**Published:** 2026-07-10

**Authors:** Bartira de Aguiar Roza, Andre Ibrahim David, Flavio Pola dos Reis, Paulo Manuel Pêgo-Fernandes

**Affiliations:** I Department of Surgical Nursing, Escola Paulista de Enfermagem, Universidade Federal de São Paulo (UNIFESP), São Paulo (SP), Brazil; WHO Expert Advisory Panel/Committee on Donation and Transplantation (ECDT). Collaborating Professor PAHO/WHO.; II Escola Paulista de Medicina, Universidade Federal de São Paulo (UNIFESP), São Paulo (SP), Brazil.; III Thoracic Surgery Department, Instituto do Coração, Hospital das Clínicas, Faculdade de Medicina, Universidade de São Paulo (USP), São Paulo (SP), Brazil.; IV Faculdade de Medicina, Faculdade de Medicina, Universidade de São Paulo (USP), São Paulo (SP), Brazil; Professor, Department of Cardiopneumology, Faculdade de Medicina, Universidade de São Paulo (USP), São Paulo (SP), Brazil; Director, Scientific Department, Associação Paulista de Medicina (APM), São Paulo (SP), Brazil.

The safe transport of substances of human origin, particularly organs and tissues for transplantation, remains one of the most sensitive and least visible aspects of the transplantation chain. Although Brazil occupies a prominent position in the global transplantation milieu, avoidable losses still occur due to logistic weaknesses that compromise graft viability, system efficiency, and above all the lives of patients on the waiting list.

Notably, a proportion of organs offered for donation are not transplanted due to problems associated with their packaging and transport, such as a lack of temperature control, risk of freezing, or excessive warming.^1^ This scenario becomes even more critical in the context of rising global temperatures linked to climate change, which increase the risk of exposure to elevated temperatures during transport, particularly over long distances or in regions with limited infrastructure. International reports issued by the World Health Organization and United Nations warn that global warming is already affecting the safety of healthcare supply chains, with the adoption of resilient strategies and secure pathways being required to preserve substances of human origin.^2,3^ These factors affect graft viability and also pose important ethical dilemmas: Each organ lost represents an opportunity denied to a patient on the waiting list for an organ or tissue.

Therefore, temperature variation during the transport of solid organs remains a major challenge, with evidence showing that both cold ischemia time and thermal stability along the route directly influence transplant outcomes.^4^

In lung transplantation, strict temperature control during graft preservation is crucial to reduce ischemic injury. Studies indicate that, compared with the conventional ice method, lungs maintained in ice-free preservation systems at a stable temperature between 6 °C and 8 °C can undergo prolonged periods of cold ischemia without worsening of clinical outcomes.^5^ Conversely, temperature fluctuations outside this range are associated with a high risk of primary graft dysfunction and reperfusion edema.

The possibility of extending safe cold ischemia time without increasing organ damage has a direct impact on transplant logistics. Such an extension would facilitate organ procurement from more distant centers, thus expanding the geographic reach of donation; it would also potentially contribute to an increase in the number of transplants performed. Furthermore, the extension of cold ischemia time would facilitate semi-elective scheduling of the transplantation, thereby optimizing intrahospital logistics, expanding the range of possible interventions such as intraoperative plasmapheresis in sensitized recipients, and providing greater predictability for recipient arrival and preparation.^6^

Initial studies in heart transplantation using temperature-controlled systems have shown promising results, enabling ischemia times to be lengthened without increasing graft dysfunction.^7^

A similar situation occurs in liver transplantation. Prolonged exposure to uncontrolled hypothermia increases the risk of ischemia-reperfusion injury. Normothermic *ex situ* perfusion has proven to be an effective strategy for mitigating the deleterious effects of temperature variations, with liver function being maintained even after prolonged periods of preservation.^8^

Therefore, traditional transport models based on insulated boxes with ice, without continuous monitoring, do not meet the requirements of a modern, quality-oriented system. Thermal variability is associated with a high risk of cell injury, poor posttransplant outcomes,^4,9^ and impaired traceability and management of adverse events.^10^

In this context, analyses of adverse events related to the donation–transplantation process must be strengthened, highlighting the role of biovigilance and quality management in ensuring the safety of substances of human origin.^1,11^ From an ethical and regulatory standpoint, the World Health Organization confirms that substances of human origin must be collected, stored, and transported under validated, safe, and traceable conditions.^12^

Therefore, adopting transport technologies with active thermal monitoring, that are aligned with international recommendations for the traceability and safety of substances of human origin, represents a technical advance as well as an ethical and public health requirement.^13^ Investing in innovation, scientific validation, and process standardization is essential to reduce losses, expand access to transplantation, and uphold the social and ethical value of donations.
